# Impact of early oral-motor intervention combined with kangaroo mother care on feeding outcomes in extremely preterm infants: Mediation by oral-motor function (NOMAS) and independent risk factor analysis for feeding intolerance

**DOI:** 10.1097/MD.0000000000049891

**Published:** 2026-08-14

**Authors:** Kunyan Zhang, Yue Chu, Hui Yang, Tong Wang, Shasha Cui

**Affiliations:** a Pediatrics Outpatient Department, Tangshan Maternal and Child Health Hospital, Tangshan, Hebei, China; b Operating Room, Tangshan Maternal and Child Health Hospital, Tangshan, Hebei, China; c Department of Neonatology, Tangshan Maternal and Child Health Hospital, Tangshan, Hebei, China.

**Keywords:** extremely preterm infants, feeding intolerance, kangaroo mother care, NOMAS, oral-motor intervention

## Abstract

Feeding intolerance (FI) and immaturity hinder nutritional advancement in extremely preterm infants. While kangaroo mother care (KMC) and early oral-motor intervention (EOMI) show individual benefits, evidence for their combined effect on FI via improved oral-motor function remains limited. This retrospective observational study included 428 infants (gestational age < 28 weeks) admitted between June 2021 and June 2024. Infants were grouped by exposure to EOMI + KMC (at least one 60-minute session by the first oral-feeding training day) versus EOMI-only. Outcomes included FI, time to 50% and 80% oral intake, and feeding efficiency. Firth penalized logistic regression and linear regression were used. Counterfactual mediation analysis assessed the role of Neonatal Oral-Motor Assessment Scale severity (0–2) as a mediator. After full adjustment, EOMI + KMC was associated with lower odds of FI (odds ratio [OR] 0.73, 95% confidence interval 0.53–0.99) and higher odds of achieving the 80% intake milestone (OR 1.43, 95% confidence interval 1.01–2.04). Among achievers, EOMI + KMC reduced the time to reach 50% (ratio 0.90) and 80% (ratio 0.89) oral intake. Feeding efficiency (mean difference + 0.18 mL/min) and intake ratio (mean difference + 0.022) were significantly higher. Mediation analysis revealed a significant natural indirect effect through Neonatal Oral-Motor Assessment Scale (OR 0.92), accounting for 26.5% of the total association. Effect modification was observed by gestational age and ventilation status. Sensitivity analyses confirmed robustness. In this retrospective observational study, EOMI combined with timely KMC was associated with improved feeding outcomes and lower odds of FI, with early oral-motor function explaining a meaningful portion of this association. These findings support integrating KMC into structured EOMI protocols.

## 1. Introduction

Feeding immaturity is a major barrier to recovery in extremely preterm infants, who often require prolonged tube feeding and are vulnerable to feeding intolerance (FI), delayed nutritional advancement, and extended hospitalization.^[1,2]^ Kangaroo mother care (KMC), defined by sustained skin-to-skin contact (SSC) with support for breastmilk feeding, is one of the most evidence-based, low-cost neonatal care strategies. In the WHO Immediate KMC Study Group multi-country randomized controlled trial conducted in 5 hospitals in Ghana, India, Malawi, Nigeria, and Tanzania (n = 3211; birth weight 1.0 to < 1.8 kg), immediate KMC significantly reduced neonatal mortality at 28 days compared with conventional care (12.0% vs 15.7%; risk ratio [RR] 0.75, 95% confidence interval [CI] 0.64–0.89).^[3]^ A complementary evidence base comes from the 2016 Cochrane review of randomized trials of KMC in stabilized low-birthweight infants, which reported higher exclusive breastfeeding at discharge or at 40 to 41 weeks postmenstrual age (66.3% vs 56.3%; RR 1.16, 95% CI 1.07–1.25) and lower severe infection/sepsis at latest follow-up (6.6% vs 13.1%; RR 0.50, 95% CI 0.36–0.69), supporting clinically meaningful benefits beyond survival.^[4]^

Alongside KMC, oral-motor interventions have been developed to accelerate the transition from tube feeding to safe oral feeding by improving sucking–swallowing coordination and perioral strength. In a meta-analysis with trial sequential analysis by Tian and colleagues pooling 11 randomized controlled trials (855 preterm infants), oral motor intervention was associated with a shorter transition time from tube feeding to 80% oral-intake milestone feeding (mean difference [MD] − 4.03 days, 95% CI − 5.22 to − 2.84) and a shorter length of hospital stay (MD − 3.64 days, 95% CI − 5.57 to − 1.71), suggesting that targeted stimulation can meaningfully shorten the feeding trajectory.^[5]^ More recently, Singh and colleagues conducted a randomized trial in a neonatal intensive care unit (NICU; n = 84; preterm neonates 29 + 0 to 33 + 6 weeks corrected gestational age [GA]) comparing structured premature infant oral motor intervention (PIOMI) with routine oromotor stimulation; they reported higher odds of exclusive breastfeeding after discharge (1 month: odds ratio [OR] 6.364, 95% CI 1.262–32.079; 3 months: OR 3.889, 95% CI 1.186–12.749) and shorter feeding progression metrics (transition time to 80% oral-intake milestone feeds 2 days lower; hospitalization 8 days lower in the PIOMI group), supporting the plausibility that modifiable oral-motor function contributes to downstream feeding outcomes.^[6]^

Despite these advances, FI remains a frequent and clinically consequential complication in preterm care, and its determinants are multifactorial. In a cohort study by Junqueira and colleagues of 251 preterm infants (<34 weeks gestation), FI occurred in 17.5% of infants; hypermagnesemia at birth was independently associated with FI (multivariable OR 2.51, 95% CI 1.06–5.91), illustrating how metabolic exposures and illness severity indicators can amplify FI risk.^[7]^ These observations underscore a persistent gap: while KMC and oral-motor stimulation each show benefits in selected preterm populations, there is limited real-world evidence addressing their combined implementation within routine NICU care, and mechanistic quantification of whether improvements in early oral-motor function transmit benefits to clinically important feeding endpoints such as FI is uncommon.^[8,9]^

While prior studies have examined KMC or oral-motor interventions independently, evidence on their combined implementation in routine NICU care and the extent to which early oral-motor function mediates FI remains limited. Our study addresses this gap by evaluating early oral-motor intervention (EOMI) + KMC versus EOMI alone in extremely preterm infants and quantifying mediation via Neonatal Oral-Motor Assessment Scale (NOMAS) severity through a record-based retrospective observational study.^[1,10]^ We further quantified whether oral-motor function at the first oral-feeding training, assessed by the NOMAS, mediated the association between combined care and FI, and we performed an independent risk factor analysis to contextualize the relative contribution of perinatal maturity and early clinical course factors to FI risk.

## 2. Methodology

### 2.1. Study design and setting

We conducted a record-based retrospective observational study in the NICU of Tangshan Maternal and Child Health Hospital (Tangshan Women and Children’s Hospital), Hebei, China. The analytic dataset was constructed at the level of one record per infant by extracting routine clinical and nursing documentation from the electronic medical record after completion of the index hospitalization (discharge or transfer) for admissions occurring between June 1, 2021 and June 30, 2024. The prespecified objectives were to compare feeding outcomes between infants exposed to EOMI and those exposed to EOMI combined with KMC (EOMI + KMC), to quantify the mediating role of oral-motor function assessed using the NOMAS, and to identify independent risk factors for FI in the same record-based dataset. Data were extracted using a standardized abstraction form by 2 trained reviewers; discrepancies were resolved by consensus with a senior neonatologist, and a random subset of records was rechecked for accuracy. Given the retrospective observational design, no formal a priori sample size calculation was undertaken; we analyzed all eligible infants during the study period with complete documentation for prespecified variables.

### 2.2. Participants

All infants admitted to the NICU during the prespecified study window were screened consecutively using the electronic medical record, and the cohort represents a consecutive census of all eligible admissions during this period. Infants were eligible if they were extremely preterm (GA < 28 completed weeks), had birth weight < 2000 g, and received intravenous therapy at admission as part of standard NICU management. Infants were excluded if they had congenital craniofacial anomalies involving the oral cavity, structural gastrointestinal obstruction, severe cardiopulmonary or other major organ dysfunction that precluded oral stimulation as documented by the attending neonatologist, a documented contraindication to oral stimulation/oral feeding, or incomplete documentation for the prespecified exposure, mediator, and primary outcome variables. To avoid duplicate observations, only the first eligible hospitalization per infant during the study period was retained. The final analytic sample comprised 428 infants, including 230 classified as EOMI-only and 198 classified as EOMI + KMC according to the prespecified exposure definition (see below), with complete data for oral-motor function at first oral feeding training (NOMAS) and FI. Baseline demographics and perinatal/clinical covariates were abstracted using a standardized data abstraction form with prespecified coding rules.

### 2.3. Exposure ascertainment and nursing procedures

EOMI was implemented as routine nursing care and recorded in the daily nursing procedure log. Each EOMI session lasted 300 seconds and followed a fixed sequence performed by trained NICU nurses wearing sterile gloves: cheek stimulation using an intrabuccal thumb with an extra-buccal index finger to massage from the oral commissure toward the cheek and back in a C-shaped trajectory (30 seconds), inner–outer lip facilitation with gentle pinching and lateral movement across the lip and return to neutral (30 seconds), perioral stimulation by tracing 1 complete circle around the mouth (30 seconds), gingival stimulation by circular massage along the external gingival margin for 2 complete circles (30 seconds), tongue-root facilitation by guiding the tongue root toward midline and returning to neutral bilaterally (15 seconds per side), alternating palate–tongue light pressure cycles with 3 seconds on the palate followed by 3 seconds on the tongue repeated for 30 seconds, sucking-reflex elicitation by gentle in-mouth finger vibration (15 seconds), and pacifier-based nonnutritive sucking practice under monitoring (120 seconds). KMC was delivered according to a standardized nursing pathway and documented in a dedicated KMC log. A completed KMC session required continuous SSC for 60 minutes with the infant (diaper and socks only) placed upright prone against the parent’s bare chest with head and trunk aligned and the airway unobstructed, secured with a wrap; nursing checks were routinely documented during the session per unit practice. Exposure status was defined a priori at the infant level: infants were classified as EOMI + KMC if the KMC log documented at least one completed 60-minute KMC session on or before the NOMAS assessment day (the first oral feeding training day); infants were classified as EOMI-only otherwise.

### 2.4. Outcomes and measurements

GA (weeks) was abstracted from the delivery record and retained as a continuous variable; GA was additionally categorized into 23 to 24, 25 to 26, and 27 weeks for descriptive reporting. Birth weight (g) was obtained from the birth record and retained as a continuous variable for modeling; for Table 1 descriptive reporting, birth weight was additionally categorized into < 750 g, 750 to 999 g, 1000 to 1249 g, and ≥ 1250 g. Sex was coded as female or male. Mode of delivery was coded as vaginal or cesarean. Perinatal asphyxia was coded as yes/no based on a documented clinical diagnosis of neonatal asphyxia in the delivery/neonatal admission record. Intrauterine infection was coded as yes/no based on a documented diagnosis of intrauterine infection/chorioamnionitis or equivalent maternal–fetal infection diagnosis recorded in obstetric/neonatal admission documentation. Patent ductus arteriosus (PDA) was coded as yes/no based on echocardiographic diagnosis recorded during hospitalization. Mechanical ventilation exposure was coded as yes/no and defined as receipt of invasive mechanical ventilation at any time during the index hospitalization.

**Table 1:** Baseline characteristics of included extremely preterm infants by exposure group (n = 428).

Characteristics	All (n = 428)	EOMI-only (n = 230)	EOMI + KMC (n = 198)
Gestational age at birth, wks (mean ± SD)	26.2 ± 0.9	26.0 ± 0.9	26.5 ± 0.8
Gestational age groups (wks)			
23–24	40 (9.3)	30 (13.0)	10 (5.1)
25–26	252 (58.9)	140 (60.9)	112 (56.6)
27	136 (31.8)	60 (26.1)	76 (38.4)
Birth weight, g (mean ± SD)	950 ± 190	910 ± 195	1000 ± 175
Birth weight groups (g)			
<750	70 (16.4)	50 (21.7)	20 (10.1)
750–999	160 (37.4)	95 (41.3)	65 (32.8)
1000–1249	140 (32.7)	65 (28.3)	75 (37.9)
≥1250	58 (13.6)	20 (8.7)	38 (19.2)
Sex, n (%)			
Female	193 (45.1)	100 (43.5)	93 (47.0)
Male	235 (54.9)	130 (56.5)	105 (53.0)
Mode of delivery, n (%)			
Vaginal	162 (37.9)	92 (40.0)	70 (35.4)
Caesarean	266 (62.1)	138 (60.0)	128 (64.6)
Perinatal asphyxia, n (%)			
Yes	86 (20.1)	55 (23.9)	31 (15.7)
No	342 (79.9)	175 (76.1)	167 (84.3)
Intrauterine infection, n (%)			
Yes	77 (18.0)	49 (21.3)	28 (14.1)
No	351 (82.0)	181 (78.7)	170 (85.9)
Patent ductus arteriosus (PDA), n (%)			
Yes	202 (47.2)	122 (53.0)	80 (40.4)
No	226 (52.8)	108 (47.0)	118 (59.6)
Mechanical ventilation during hospitalization, n (%)			
Yes	238 (55.6)	150 (65.2)	88 (44.4)
No	190 (44.4)	80 (34.8)	110 (55.6)
Oral-motor function at first oral feeding training (NOMAS pattern), n (%)			
Normal (severity = 0)	150 (35.0)	68 (29.6)	82 (41.4)
Disorganized (severity = 1)	198 (46.3)	112 (48.7)	86 (43.4)
Dysfunctional (severity = 2)	80 (18.7)	50 (21.7)	30 (15.2)

Values are mean ± SD for continuous variables and n (column %) for categorical variables. Exposure was defined as EOMI + KMC if the KMC log documented at least one completed 60-minute KMC session on or before the NOMAS assessment day (the first oral feeding training day); otherwise, infants were classified as EOMI-only. NOMAS pattern was recorded on the first oral feeding training day and coded as an ordinal severity score (0 = normal, 1 = disorganized, and 2 = dysfunctional) for regression/mediation analyses. Gestational age and birth weight were modeled as continuous covariates in regression models but are additionally shown as categorical strata for descriptive reporting. All infants received intravenous therapy at admission by eligibility. Percentages may not sum to 100 due to rounding.

EOMI = early oral-motor intervention, KMC = kangaroo mother care, NOMAS = Neonatal Oral-Motor Assessment Scale.

Oral-motor function (the prespecified mediator) was assessed using NOMAS on the first calendar day when an oral feeding training attempt was documented in the feeding chart, and the NOMAS pattern classification (normal, disorganized, and dysfunctional) was recorded by trained staff.^[11,12]^ For regression and mediation analyses, NOMAS was operationalized as an ordinal severity score coded 0 (normal), 1 (disorganized), and 2 (dysfunctional), with higher values indicating poorer oral-motor function. Feeding skill acquisition and feeding outcomes were derived from routine feeding charts using standardized calculations. For each infant-day, the prescribed 24-hour target milk volume was the neonatologist-ordered total milk volume documented in the daily nutrition plan (mL/kg/d multiplied by recorded body weight for that day).^[13]^ The daily oral intake proportion was calculated as total oral milk volume over 24 hours divided by the prescribed 24-hour target volume. The 50% milestone was defined as an oral intake proportion ≥ 0.50 sustained for 48 consecutive hours, and the 80% milestone was defined as an oral intake proportion ≥ 0.80 sustained for 48 consecutive hours. Time to each milestone was counted in days from the first oral feeding training day (the NOMAS assessment day) to the first day of the qualifying 48-hour window; infants who did not achieve a milestone before discharge were coded as “not achieved” for milestone attainment endpoints. Because exposure was defined based on completed KMC sessions occurring on or before the NOMAS assessment day, temporal ordering for mediation was ensured by design.^[14,15]^

Feeding efficiency, feeding effectiveness, and intake ratio were operationalized a priori from the same routine feeding documentation. Feeding efficiency (mL/min) was defined as the total oral milk volume (mL) consumed divided by the total active oral-feeding time (minutes) recorded by nurses, calculated over the first 24 hours after the 50% milestone was first achieved; for infants who did not achieve the 50% milestone, feeding efficiency was calculated over the first 24 hours starting from the NOMAS assessment day using the same formula. Feeding effectiveness was defined as the proportion of planned oral-feeding sessions completed without interruption due to clinical instability between the NOMAS assessment day and discharge, with interruption recorded when a session was stopped for oxygen desaturation, bradycardia, apnea, or vomiting requiring cessation as documented in the nursing feed record; the resulting proportion ranged from 0 to 1 for each infant.^[16,17]^ Intake ratio (actual/prescribed) was defined as the infant’s actual 24-hour total milk intake (oral plus tube feeding) divided by the prescribed 24-hour target milk volume, calculated on the day the 80% milestone was first achieved; if the 80% milestone was not achieved, the intake ratio was calculated on the last hospital day.^[18,19]^

FI was adjudicated from daily nursing notes and medical orders during feeding advancement and was defined as clinician-directed reduction or interruption of milk feeding attributed to intolerance accompanied by at least one objective criterion: gastric residual volume ≥ 50% of the immediately preceding feeding volume on 2 consecutive pre-feed aspirations, vomiting ≥ 2 episodes within 24 hours, abdominal distension defined as an increase in abdominal circumference ≥ 2 cm within 24 hours compared with the prior day together with a decision to reduce or hold feeds, or feed interruption for ≥ 12 hours or feed-volume reduction ≥ 20% sustained for ≥ 24 hours due to intolerance.^[7,20,21]^ FI was coded as present if any criterion occurred at least once during hospitalization.

### 2.5. Candidate predictors for FI

Candidate predictors for FI were prespecified a priori and extracted uniformly from the medical record, including GA (weeks), birth weight (g), sex (female/male), mode of delivery (vaginal/cesarean), perinatal asphyxia (yes/no), intrauterine infection (yes/no), PDA (yes/no), and mechanical ventilation during hospitalization (yes/no). Predictors were coded as defined in Table 1, with GA and birth weight retained on their original continuous scales for regression modeling; for descriptive reporting only, GA and birth weight were additionally presented in clinically meaningful categories in Table 1. Exposure status (EOMI + KMC vs EOMI-only) was included as the main independent variable in all primary models and was also entered as a covariate in FI risk factor models.

### 2.6. Statistical analysis

Descriptive statistics were first presented for the overall participants and by exposure group (EOMI-only vs EOMI + KMC). Continuous variables were summarized as mean (standard deviation) when approximately normally distributed and as median (interquartile range) otherwise; categorical variables were summarized as number (percentage). Normality was assessed using the Shapiro–Wilk test. Group comparisons used the Welch *t*-test for approximately normal continuous variables, the Wilcoxon rank-sum test for non-normal continuous variables, and the χ^2^ test for categorical variables, with Fisher exact test applied when any expected cell count was < 5.

Covariates were prespecified based on clinical relevance and prior evidence to capture key domains of maturity (GA and birthweight), perinatal factors (mode of delivery, asphyxia, and intrauterine infection), and illness severity during hospitalization (PDA and mechanical ventilation).^[22–25]^ Missing data were handled using complete-case analysis when missingness for any analysis variable was ≤ 5%; when missingness exceeded 5%, pooled estimates were generated using Rubin rules after multiple imputation by chained equations (20 datasets). Primary exposure–outcome associations were estimated using prespecified multivariable models with 4 nested adjustment sets. For each endpoint, Model I included exposure only; Model II additionally adjusted for GA, birth weight, and sex; Model III additionally adjusted for mode of delivery and perinatal asphyxia; and Model IV (primary) additionally adjusted for PDA, mechanical ventilation, and intrauterine infection. FI and achievement of the 80% oral intake milestone before discharge were analyzed as binary outcomes using logistic regression with Firth penalized likelihood, reporting ORs and 95% CIs. Time to the 50% milestone and time to the 80% milestone (days) were analyzed among infants who achieved the corresponding milestone (as defined by the prespecified 48-hour sustained criteria) after natural-log transformation of days; linear regression was fitted on the log-days scale, and exponentiated coefficients were reported as ratios of geometric means (95% CIs), with values < 1 indicating shorter time in the EOMI + KMC group. Feeding efficiency (mL/min) and intake ratio (actual/prescribed) were analyzed using linear regression with heteroskedasticity-consistent robust standard errors and reported as MDs (EOMI + KMC minus EOMI-only) with 95% CIs. Feeding effectiveness (0–1) was analyzed on the logit-transformed scale log([*P* + .005]/[1 − *P* + .005]), where *P* denotes the observed effectiveness proportion, using linear regression with heteroskedasticity-consistentrobust standard errors and reported as β (95% CI) on the logit scale. Model diagnostics included residual plots for linear models and influence assessment using Cook distance (threshold 4/n).

Effect modification was evaluated by adding prespecified exposure-by-subgroup interaction terms to Model IV for each candidate modifier. Subgroup-specific effect estimates were reported for all prespecified subgroups to improve transparency, while *P* values for interaction were used to assess statistical evidence of effect modification. For each subgroup analysis, Model IV was refitted within each stratum to obtain stratum-specific estimates, adjusting for the full Model IV covariate set while excluding the stratifying variable where applicable.

After fitting the primary models, mediation by oral-motor function was examined under the counterfactual framework, treating exposure (EOMI + KMC vs EOMI-only) as the independent variable, NOMAS severity (0–2) as the mediator, and FI as the primary outcome. For mediation analyses, FI was defined as FI events occurring on or after the NOMAS assessment day (the first oral feeding training day) to preserve temporal ordering. The mediator model regressed NOMAS severity on exposure and the Model IV covariates using linear regression. The outcome model regressed FI on exposure, NOMAS severity, and the same Model IV covariates using logistic regression with Firth penalized likelihood. Natural direct effects (NDE), natural indirect effects (NIE), and total effects (TE) were estimated on the odds-ratio scale using nonparametric bootstrap with 5000 resamples; the proportion mediated was calculated on the log(OR) scale as log(OR_NIE)/log(OR_TE), with percentile 95% CIs derived from the bootstrap distribution.

Independent risk factors for FI were evaluated by fitting univariable models for each prespecified predictor, followed by a multivariable FI model including all prespecified predictors and exposure status; FI models were estimated using logistic regression with Firth penalized likelihood and reported as adjusted ORs with 95% CIs. Model discrimination was quantified using the area under the receiver operating characteristic curve, and calibration was assessed using calibration intercept and calibration slope estimated by regressing observed FI status on the logit of predicted probabilities.

Sensitivity analyses were prespecified to assess robustness of the primary findings: reestimating the primary FI and 80% milestone models using standard maximum-likelihood logistic regression instead of Firth penalized likelihood; repeating primary analyses after excluding infants who received mechanical ventilation; repeating primary analyses after restricting to infants with NOMAS recorded within 48 hours of the first oral-feeding training documentation; redefining FI using the stricter criterion and refitting Model IV; and repeating complete-case analyses using multiple imputation by chained equations (20 datasets) for even variables with < 5% missingness and pooling estimates with Rubin rules.

All tests were two-sided with statistical significance defined as *P* < .05, and all analyses were performed in R (version 4.0.3).

### 2.7. Ethics

The study protocol was reviewed and approved by the institutional ethics committee of Tangshan Maternal and Child Health Hospital (Institutional Review Board No.: TSFY-LL-JL-030-03). Written informed consent for the use of clinical and nursing data for research was obtained from a parent or legal guardian. All extracted data were de-identified prior to analysis and stored on access-restricted systems in accordance with institutional policies.

## 3. Results

### 3.1. Characteristics of included participants

The final analytic sample comprised 428 extremely preterm infants, including 230 (53.7%) classified as EOMI-only and 198 (46.3%) classified as EOMI + KMC (Table 1). Compared with the EOMI-only group, infants in the EOMI + KMC group had a slightly higher mean GA (26.5 ± 0.8 vs 26.0 ± 0.9 weeks) and higher mean birth weight (1000 ± 175 vs 910 ± 195 g). Consistent with these differences, the EOMI + KMC group contained fewer infants born at 23 to 24 weeks (5.1% vs 13.0%) and fewer with birth weight < 750 g (10.1% vs 21.7%), but a greater proportion with birth weight ≥ 1250 g (19.2% vs 8.7%). Sex distributions were broadly similar between groups, with females accounting for 47.0% of the EOMI + KMC group and 43.5% of the EOMI-only group, and mode of delivery was comparable, although cesarean delivery was slightly more frequent in the EOMI + KMC group (64.6% vs 60.0%). Markers of perinatal and early clinical complexity were less common among infants who received KMC: perinatal asphyxia (15.7% vs 23.9%), intrauterine infection (14.1% vs 21.3%), PDA (40.4% vs 53.0%), and mechanical ventilation exposure (44.4% vs 65.2%) were all lower in the EOMI + KMC group than in the EOMI-only group. Oral-motor function at first oral feeding training also differed by exposure group, with a higher proportion of normal NOMAS patterns in the EOMI + KMC group (41.4% vs 29.6%) and a lower proportion of dysfunctional patterns (15.2% vs 21.7%), while disorganized patterns remained common in both groups (43.4% vs 48.7%). Overall, these baseline differences suggest that infants who received KMC tended to have slightly greater maturity at birth and a less intensive clinical course, alongside more favorable oral-motor profiles at the time of initiating oral feeding training (Table 1).

### 3.2. Associations of EOMI + KMC with feeding outcomes

Table 2 summarizes the associations of EOMI + KMC versus EOMI-only (reference) with binary and continuous feeding endpoints across 4 prespecified models. After full adjustment (Model IV), EOMI + KMC was associated with lower odds of FI (OR = 0.73, 95% CI 0.53–0.99) and higher odds of achieving the 80% oral-intake milestone before discharge (OR = 1.43, 95% CI 1.01–2.04). EOMI + KMC was also associated with faster milestone attainment among infants who achieved the corresponding milestone, with ratios of geometric means of 0.90 (95% CI 0.84–0.97) for time to the 50% milestone and 0.89 (95% CI 0.82–0.97) for time to the 80% milestone, indicating shorter times relative to EOMI-only. For feeding performance metrics, EOMI + KMC showed a modest increase in feeding efficiency in Model IV (MD + 0.18 mL/min, 95% CI 0.02–0.34) and a higher intake ratio (MD + 0.022, 95% CI 0.002–0.042), whereas the association with feeding effectiveness on the logit scale was positive but not statistically significant after full adjustment (β = 0.22, 95% CI − 0.03 to 0.47). Across sequential models, effect estimates attenuated with covariate adjustment while remaining directionally consistent, with the FI OR increasing from 0.47 in Model I to 0.73 in Model IV, the 80% milestone OR decreasing from 1.86 to 1.43, and the time-ratio estimates increasing from 0.84 to 0.90 for the 50% milestone and from 0.82 to 0.89 for the 80% milestone (Table 2).

**Table 2:** Associations of EOMI + KMC versus EOMI-only with feeding outcomes (n = 428).

Outcomes (effect measure)	Model I	Model II	Model III	Model IV (primary)
Feeding intolerance (FI; Yes: n = 120), OR (95% CI)	0.47 (0.31–0.74)	0.58 (0.38–0.89)	0.63 (0.41–0.97)	0.73 (0.53–0.99)
Achieved 80% oral-intake milestone before discharge (Yes: n = 270), OR (95% CI)	1.86 (1.24–2.78)	1.55 (1.03–2.34)	1.49 (0.99–2.25)	1.43 (1.01–2.04)
Time to 50% oral-intake milestone (Achieved: n = 370), ratio of geometric means (95% CI)	0.84 (0.78–0.90)	0.87 (0.81–0.94)	0.88 (0.82–0.95)	0.90 (0.84–0.97)
Time to 80% oral-intake milestone (Achieved: n = 270), ratio of geometric means (95% CI)	0.82 (0.76–0.89)	0.86 (0.79–0.94)	0.87 (0.80–0.95)	0.89 (0.82–0.97)
Feeding efficiency (mL/min; n = 428), mean difference (95% CI)	+0.28 (0.12–0.44)	+0.24 (0.08–0.40)	+0.22 (0.06–0.38)	+0.18 (0.02–0.34)
Feeding effectiveness (0–1; n = 428), β on logit scale (95% CI)	0.36 (0.10–0.62)	0.30 (0.03–0.57)	0.28 (0.01–0.55)	0.22 (−0.03–0.47)
Intake ratio (actual/prescribed; n = 428), mean difference (95% CI)	+0.032 (0.012–0.052)	+0.028 (0.008–0.048)	+0.026 (0.006–0.046)	+0.022 (0.002–0.042)

All effect estimates compare EOMI + KMC vs EOMI-only (reference group), where EOMI + KMC exposure was defined as ≥ 1 completed 60-minute KMC session documented in the KMC log on or before the NOMAS assessment day (the first oral feeding training day). Binary endpoints (FI; achieved 80% milestone) were analyzed using Firth penalized logistic regression and are reported as odds ratios (ORs) with 95% confidence intervals (CIs). Time-to-milestone endpoints were analyzed among infants who achieved the corresponding milestone on the natural-log (days) scale using linear regression and are reported as ratios of geometric means; values < 1 indicate shorter time (faster attainment) in the EOMI + KMC group. Feeding efficiency and intake ratio were analyzed using linear regression and are reported as mean differences (EOMI + KMC minus EOMI-only). Feeding effectiveness (0–1) was analyzed on the logit-transformed scale and is reported as β on the logit scale; positive values indicate higher effectiveness in the EOMI + KMC group. Model definitions: Model I unadjusted; Model II adjusted for gestational age (weeks), birth weight (g), and sex; Model III additionally adjusted for mode of delivery (vaginal/cesarean) and perinatal asphyxia (yes/no); and Model IV (primary) additionally adjusted for patent ductus arteriosus (yes/no), mechanical ventilation during hospitalization (yes/no), and intrauterine infection (yes/no).

EOMI = early oral-motor intervention, KMC = kangaroo mother care, NOMAS = Neonatal Oral-Motor Assessment Scale.

### 3.3. Mediation by oral-motor function

**Figure 1.**
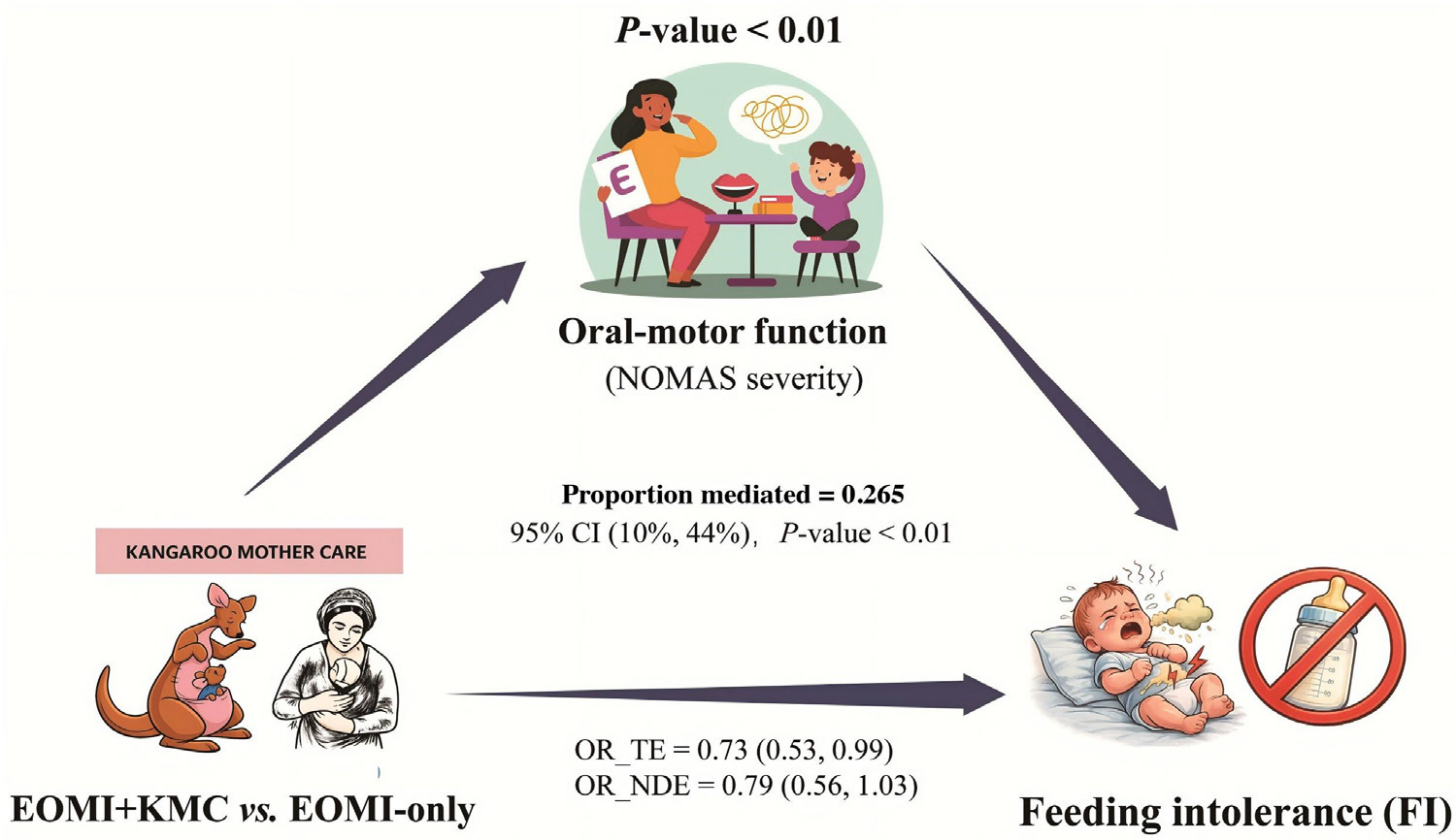
Mediation pathway of oral-motor function (NOMAS) linking EOMI + KMC to feeding intolerance (FI). This diagram illustrates the counterfactual mediation framework with exposure defined as EOMI + KMC versus EOMI-only, where EOMI + KMC indicates at least one completed 60-minute kangaroo mother care session documented in the KMC log on or before the NOMAS assessment day (the first oral-feeding training day). The mediator is oral-motor function at first oral-feeding training, operationalized as NOMAS severity (0 = normal, 1 = disorganized, and 2 = dysfunctional; higher values indicate poorer function). The outcome is feeding intolerance (FI; binary), restricted in mediation analyses to FI events occurring on or after the NOMAS assessment day to preserve temporal ordering. The total effect (OR_TE = 0.73, 95% CI 0.53–0.99) was decomposed into a natural indirect effect through NOMAS (OR_NIE = 0.92, 95% CI 0.86–0.98; proportion mediated = 0.265, 95% CI 0.10–0.44) and a natural direct effect not operating through NOMAS (OR_NDE = 0.79, 95% CI 0.56–1.03). All models adjusted for Model IV covariates (gestational age, birth weight, sex, mode of delivery, perinatal asphyxia, PDA, mechanical ventilation, and intrauterine infection). Confidence intervals were obtained using 5000 bootstrap resamples. CI = confidence interval, EOMI = early oral-motor intervention, KMC = kangaroo mother care, NDE = natural direct effect, NIE = natural indirect effect, NOMAS = Neonatal Oral-Motor Assessment Scale, ORs = odds ratios, PDA = patent ductus arteriosus.

The mediation analysis (n = 428) supported oral-motor function as a meaningful pathway linking EOMI + KMC to feeding outcomes, indicating that part of the protective association with FI operates through improved NOMAS severity at the first oral-feeding training. Specifically, the NIE of EOMI + KMC on FI operating through NOMAS was statistically significant (OR for the NIE, OR_NIE = 0.92, 95% CI 0.86–0.98), corresponding to a mediation proportion of 26.5% (proportion mediated = 0.265, 95% CI 0.10–0.44), consistent with partial mediation. The remaining association was attributable to the NDE of EOMI + KMC on FI not operating through NOMAS (OR for the NDE, OR_NDE = 0.79, 95% CI 0.56–1.03). The TE was aligned with the fully adjusted primary model for FI (OR for the TE, OR_TE = 0.73, 95% CI 0.53–0.99). Collectively, these results suggest that EOMI + KMC reduces FI partly by improving early oral-motor function, with additional non-oral-motor pathways contributing to the residual direct component (Fig. 1).

### 3.4. Subgroup analysis for EOMI + KMC and FI

**Figure 2.**
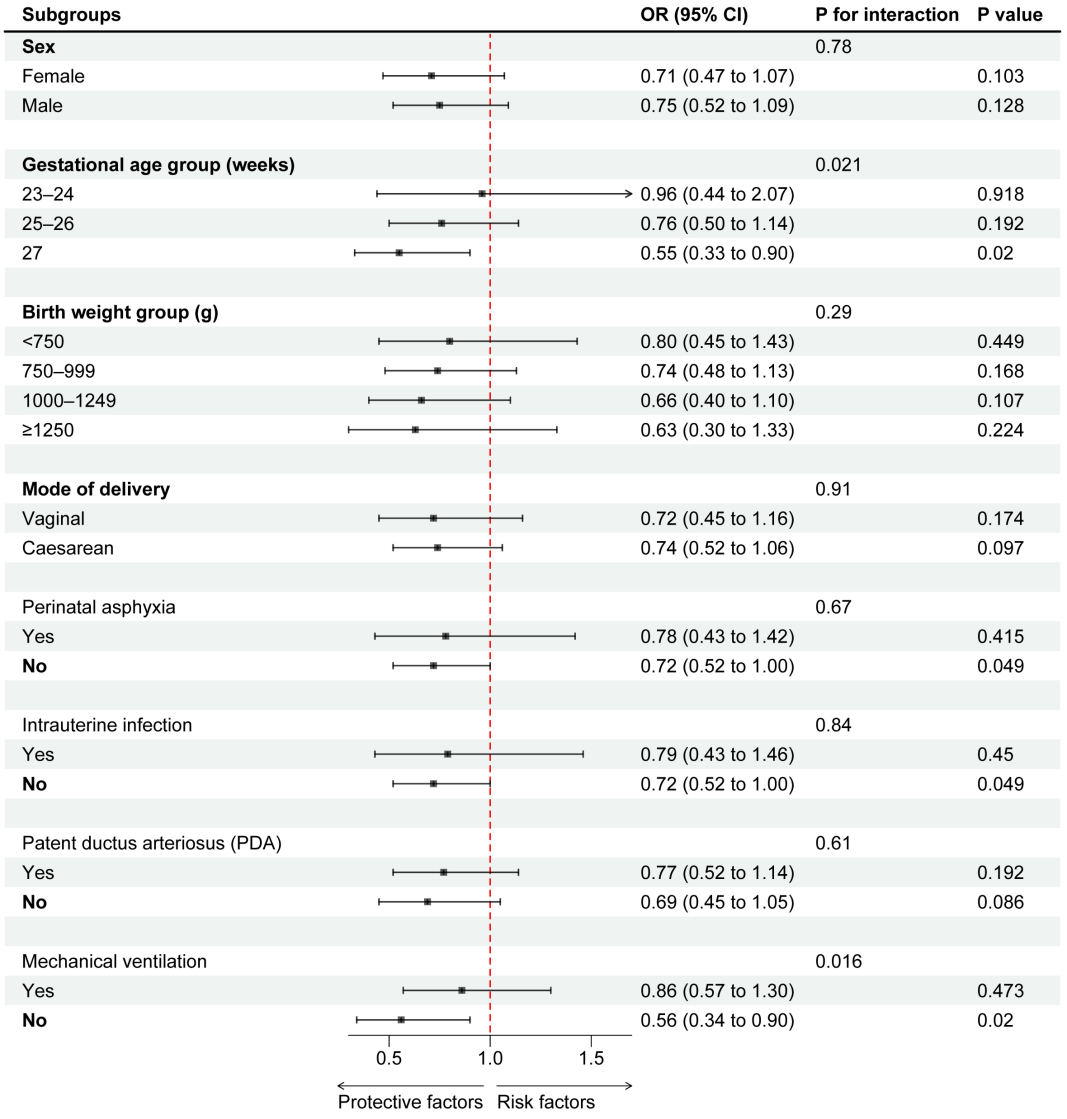
Forest plot for the subgroup analyses of the association between EOMI + KMC (vs EOMI-only) and feeding intolerance (Model IV; n = 428). Within each subgroup, models adjusted for the full Model IV covariate set (gestational age, birth weight, sex, mode of delivery, perinatal asphyxia, patent ductus arteriosus, mechanical ventilation, and intrauterine infection), excluding the stratifying variable when applicable. CI = confidence interval, EOMI = early oral-motor intervention, KMC = kangaroo mother care, ORs = odds ratios, PDA = patent ductus arteriosus.

Effect modification was explored for prespecified neonatal characteristics with respect to the association between EOMI + KMC (vs EOMI-only) and FI. In fully adjusted models (Model IV), a statistically significant interaction was observed for GA group and mechanical ventilation, whereas interactions with sex, birth weight group, mode of delivery, perinatal asphyxia, intrauterine infection, and PDA were not statistically significant (all *P* for interaction ≥ .05). Specifically, the protective association of EOMI + KMC with FI appeared stronger among infants born at 27 weeks (OR = 0.55, 95% CI 0.33–0.90) than among those born at 25 to 26 weeks (OR = 0.76, 95% CI 0.50–1.14) or 23 to 24 weeks (OR = 0.96, 95% CI 0.44–2.07; *P* for interaction = .021). In addition, the association was stronger among infants without mechanical ventilation (OR = 0.56, 95% CI 0.34–0.90) than among those who received mechanical ventilation (OR = 0.86, 95% CI 0.57–1.30; *P* for interaction = .016). Stratum-specific estimates for other modifiers were directionally consistent with the overall effect and showed no evidence of statistically significant interaction (Fig. 2, Table 3).

**Table 3:** Subgroup analyses of the association between EOMI + KMC (vs EOMI-only) and feeding intolerance (Model IV; n = 428).

Subgroup	Category	FI events/ n (%)	OR (95% CI) for EOMI + KMC vs EOMI-only	*P* for interaction
Sex	Female	56/193 (29.0)	0.71 (0.47–1.07)	.78
	Male	64/235 (27.2)	0.75 (0.52–1.09)	
Gestational age group (wks)	23–24	18/40 (45.0)	0.96 (0.44–2.07)	.021
	25–26	72/252 (28.6)	0.76 (0.50–1.14)	
	27	30/136 (22.1)	0.55 (0.33–0.90)	
Birth weight group (g)	<750	30/70 (42.9)	0.80 (0.45–1.43)	.29
	750–999	50/160 (31.3)	0.74 (0.48–1.13)	
	1000–1249	30/140 (21.4)	0.66 (0.40–1.10)	
	≥1250	10/58 (17.2)	0.63 (0.30–1.33)	
Mode of delivery	Vaginal	46/162 (28.4)	0.72 (0.45–1.16)	.91
	Caesarean	74/266 (27.8)	0.74 (0.52–1.06)	
Perinatal asphyxia	Yes	32/86 (37.2)	0.78 (0.43–1.42)	.67
	No	88/342 (25.7)	0.72 (0.52–1.00)	
Intrauterine infection	Yes	28/77 (36.4)	0.79 (0.43–1.46)	.84
	No	92/351 (26.2)	0.72 (0.52–1.00)	
Patent ductus arteriosus (PDA)	Yes	70/202 (34.7)	0.77 (0.52–1.14)	.61
	No	50/226 (22.1)	0.69 (0.45–1.05)	
Mechanical ventilation	Yes	85/238 (35.7)	0.86 (0.57–1.30)	.016
	No	35/190 (18.4)	0.56 (0.34–0.90)	

Stratum-specific estimates are from the fully adjusted model (Model IV) within each subgroup and are reported as odds ratios (ORs) with 95% confidence intervals (CIs) for EOMI + KMC vs EOMI-only (reference). Within each subgroup, models adjusted for the full Model IV covariate set (gestational age, birth weight, sex, mode of delivery, perinatal asphyxia, patent ductus arteriosus, mechanical ventilation, and intrauterine infection), excluding the stratifying variable when applicable. *P* values for interaction were obtained by adding the exposure-by-subgroup interaction term(s) to Model IV.

EOMI = early oral-motor intervention, KMC = kangaroo mother care.

### 3.5. Independent risk factors for FI

Overall, 120 of 428 infants (28.0%) met the prespecified criteria for FI. In univariable analyses, lower GA and lower birth weight, perinatal asphyxia, intrauterine infection, PDA, and mechanical ventilation were each associated with higher odds of FI, while exposure to EOMI + KMC was associated with lower odds of FI. In the multivariable FI model including all prespecified predictors and exposure status, markers of maturity and illness severity remained the dominant independent correlates of FI. Specifically, each 1-week increase in GA was associated with lower odds of FI (adjusted OR = 0.76, 95% CI 0.65–0.88) and each 100-g increase in birth weight was similarly protective (adjusted OR = 0.90, 95% CI 0.85–0.96). Mechanical ventilation was independently associated with higher FI risk (adjusted OR = 2.12, 95% CI 1.40–3.23), as were PDA (adjusted OR = 1.45, 95% CI 1.01–2.09), intrauterine infection (adjusted OR = 1.61, 95% CI 1.04–2.48), and perinatal asphyxia (adjusted OR = 1.47, 95% CI 1.00–2.18). After accounting for these covariates, EOMI + KMC retained an independent protective association with FI (adjusted OR = 0.73, 95% CI 0.53–0.99), whereas sex and mode of delivery were not independently associated with FI in the fully adjusted model. Model discrimination was acceptable, with an area under the receiver operating characteristic curve of 0.79 (95% CI 0.75–0.83), and calibration was satisfactory (calibration intercept − 0.03, calibration slope 0.97), indicating good agreement between predicted and observed FI probabilities (Table 4).

**Table 4:** Independent predictors of feeding intolerance.

Predictor	Univariable OR (95% CI)	Multivariable adjusted OR (95% CI)
Gestational age (per 1-wk increase)	0.67 (0.58–0.78)	0.76 (0.65–0.88)
Birth weight (per 100-g increase)	0.86 (0.81–0.92)	0.90 (0.85–0.96)
Male (vs female)	0.91 (0.63–1.32)	1.08 (0.74–1.58)
Caesarean (vs vaginal)	0.97 (0.66–1.44)	0.93 (0.63–1.37)
Perinatal asphyxia (yes vs no)	1.71 (1.06–2.75)	1.47 (1.00–2.18)
Intrauterine infection (yes vs no)	1.61 (0.97–2.67)	1.61 (1.04–2.48)
PDA (yes vs no)	1.87 (1.25–2.80)	1.45 (1.01–2.09)
Mechanical ventilation (yes vs no)	2.46 (1.57–3.86)	2.12 (1.40–3.23)
EOMI + KMC (vs EOMI-only)	0.47 (0.31–0.74)	0.73 (0.53–0.99)

Model performance (multivariable model). AUC = 0.79 (95% CI 0.75–0.83); calibration intercept = −0.03; calibration slope = 0.97. Odds ratios (ORs) are from firth penalized logistic regression. Gestational age and birth weight were modeled as continuous variables; ORs are reported per 1-week and per 100-g increments for interpretability.

CI = confidence interval, EOMI = early oral-motor intervention, KMC = kangaroo mother care, PDA = patent ductus arteriosus.

### 3.6. Sensitivity analyses

Five prespecified sensitivity analyses supported the robustness of the primary findings. First, replacing Firth penalized logistic regression with standard maximum-likelihood logistic regression yielded effect estimates that closely matched the primary results. In fully adjusted models (Model IV), EOMI + KMC remained associated with lower odds of FI (OR = 0.72, 95% CI 0.52–0.99) and higher odds of achieving the 80% oral-intake milestone before discharge (OR = 1.41, 95% CI 1.00–2.00). Second, after excluding infants who received mechanical ventilation (n = 190; FI events = 35), the protective association of EOMI + KMC with FI persisted and appeared stronger (Model IV: OR = 0.56, 95% CI 0.34–0.90). In the same restricted sample, EOMI + KMC was also associated with higher odds of achieving the 80% oral-intake milestone (OR = 1.52, 95% CI 1.01–2.33). Third, restricting analyses to infants with NOMAS recorded within 48 hours of the first documented oral-feeding training attempt (n = 402; FI events = 112) produced estimates consistent with the main analysis (Model IV: FI OR = 0.75, 95% CI 0.54–1.04; 80% milestone OR = 1.40, 95% CI 0.99–1.99). Fourth, applying a stricter FI definition (requiring clinician-directed feeding modification together with at least 2 concurrent intolerance signs) reduced the number of FI events (stricter FI events = 84/428, 19.6%) but yielded a similar direction of association, with EOMI + KMC remaining protective after full adjustment (Model IV: OR = 0.76, 95% CI 0.54–1.06). Fifth, multiple imputation by chained equations (20 datasets) produced pooled estimates closely aligned with complete-case results (Model IV pooled: FI OR = 0.74, 95% CI 0.55–1.00; 80% milestone OR = 1.44, 95% CI 1.02–2.05). Collectively, alternative estimation methods, restriction to lower-acuity infants, tighter mediator timing, stricter outcome definition, and imputation-based missing-data handling did not materially alter the overall conclusions.

## 4. Discussion

In this record-based retrospective observational study of extremely preterm infants, exposure to EOMI combined with KMC (documented on or before the first oral-feeding training day) was associated with a clinically coherent pattern of improved feeding outcomes. In fully adjusted models, EOMI + KMC appeared to be associated with lower odds of FI, higher odds of achieving the 80% oral-intake milestone before discharge, shorter time to milestone attainment among infants who achieved the corresponding milestone, and modest improvements in feeding efficiency and intake ratio. Mediation analysis suggested partial mediation through early oral-motor function as assessed by NOMAS severity, indicating that differences in NOMAS severity at the first oral-feeding training may account for a measurable portion of the observed association with FI, while acknowledging that residual confounding and measurement limitations may remain given the retrospective observational design. In parallel, the FI risk-factor model identified maturity and illness-severity markers (GA, birth weight, respiratory support, PDA, infection, and asphyxia) as dominant correlates of FI risk and demonstrated acceptable discrimination and calibration, suggesting potential utility for pragmatic risk stratification in routine NICU care rather than establishing causal determinants.

Our feeding-outcome findings align with and extend prior interventional evidence for KMC and oral-motor stimulation. In a randomized trial of 168 preterm infants (84 per arm), Çaka and colleagues applied 1 hour of KMC after feeding and reported significantly fewer FI events defined by abdominal distension or vomiting plus residual criteria, with 4/84 in the KMC group versus 18/84 in the standard care group (χ^2^ = 10.252, *P* = .001), alongside markedly lower gastric residual volume before the next feeding (0.84 ± 3.28 vs 4.17 ± 6.04 mL; *P* < .001) and shorter time to full enteral feeding (7.82 ± 5.30 vs 10.04 ± 8.21 days; *P* = .039).^[9]^ In another randomized trial enrolling 50 premature infants (GA 27–36 weeks, birth weight ≥ 1000 g), Şimşek et al found that kangaroo care reduced the transition period from gavage to 80% oral-intake milestone (13.60 ± 6.83 vs 22.10 ± 7.38 days; *P* = .007) and shortened time to 80% oral-intake milestone after birth (29.20 ± 8.06 vs 44.60 ± 21.90 days).^[8]^ On the oral-motor intervention side, Tian et al pooled 11 randomized trials (n = 855) in a meta-analysis with trial sequential analysis and reported that oral motor intervention reduced the transition time from tube to total oral feeding by 4.03 days (MD − 4.03; 95% CI − 5.22 to − 2.84) and shortened hospital stay by 3.64 days (MD − 3.64; 95% CI − 5.57 to − 1.71), while also increasing feeding efficiency and milk intake (MD 0.08 and MD 0.14, respectively).^[5]^ Consistently, Lessen pilot randomized, blinded clinical trial (n = 19; 26–29 weeks postmenstrual age) found that once-daily PIOMI led to transition from first oral feeding to total oral feedings 5 days sooner (*P* = .043) and discharge 2.6 days sooner than sham.^[26]^ In a more recent randomized trial (n = 84; 29 + 0–33 + 6 weeks corrected GA), Singh et al reported higher odds of exclusive breastfeeding with PIOMI at 1 month (OR 6.364; 95% CI 1.262–32.079) and 3 months (OR 3.889; 95% CI 1.186–12.749) after discharge, highlighting downstream feeding benefits from structured oral-motor stimulation.^[6]^ Against this backdrop, our combined-care associations are directionally consistent with both KMC and oral-motor intervention trials, while our FI risk-factor findings mirror broader observational evidence that FI is strongly linked to maturity and illness burden. For example, in a cohort study of preterm infants, Junqueira et al reported that hypermagnesemia was independently associated with FI (OR 2.51; 95% CI 1.06–5.91), underscoring the role of systemic vulnerability and comorbid exposures in FI risk.^[7]^ In a very low birthweight observational study (n = 132), Boo et al found that each hour delay in initiating the first feed increased FI odds (adjusted OR 1.03; 95% CI 1.01–1.05; *P* = .01), reinforcing that feeding progression is sensitive to both physiologic readiness and clinical decision-making.^[20]^ A recent extremely low birthweight cohort analysis by Soffer et al reported FI in 58% of 133 infants and showed that FI was associated with longer time to full enteral feeding (median 18 vs 14 days; *P* < .001) and longer NICU stay (median 89.5 vs 47 days; *P* < .001), emphasizing the clinical consequences of FI and its coupling with respiratory support context (higher FI rates per device-days on nasal intermittent positive pressure ventilation).^[27]^

The mediation analysis provides a mechanistic perspective that is rarely quantified in the neonatal feeding literature. We observed evidence of partial mediation, with NOMAS severity at the first oral-feeding training accounting for a measurable portion of the association between EOMI + KMC and FI, suggesting that early oral-motor organization may be 1 pathway linking combined care to feeding tolerance and progression. This interpretation is supported by intervention studies showing that oral-motor programs can improve feeding-process measures and downstream feeding outcomes, including improved feeding efficiency and milk intake with shorter transition time in the meta-analysis by Tian et al^[5]^ and higher odds of exclusive breastfeeding after discharge in Singh trial.^[6]^ However, NOMAS is not a complete surrogate for feeding progression; Bingham et al reported that NOMAS scores did not predict feeding outcomes in their prospective NICU study, underscoring the importance of maturity and broader physiologic factors.^[28]^ Taken together, these findings are consistent with partial mediation and suggest that additional NOMAS-independent pathways likely contribute to the remaining association with FI and milestone achievement.

Several physiologic mechanisms could plausibly connect KMC plus oral-motor stimulation to be associated with improved feeding tolerance. SSC has been linked to enhanced autonomic regulation and stress resilience, which can influence gastrointestinal motility and perfusion. In a cohort study of 101 preterm neonates with weekly ECG monitoring, Marvin et al reported that at postnatal week 1, SSC frequency and high-frequency heart rate variability (a marker of vagal tone) were positively correlated (*P* = .02), and in the highest-morbidity subgroup SSC frequency was associated with higher vagal tone (*r*_s = 0.382, *P* = .004) and shorter length of stay (*r*_s=−0.419, *P* = .001).^[29]^ A meta-analysis of randomized trials evaluating physiologic stress parameters found that KMC reduced respiratory rate by a MD of − 3.50 breaths/min (95% CI − 5.17 to − 1.83; *P* < .001), suggesting improved physiologic stability during or after KMC exposure.^[30]^

Gastrointestinal and biochemical pathways may also contribute to the observed associations. At the gastrointestinal level, the randomized trial by Çaka et al demonstrated lower gastric residual volume (0.84 ± 3.28 vs 4.17 ± 6.04 mL) and fewer FI events in the KMC group, supporting a link between KMC positioning/parasympathetic tone and improved gastric emptying or reduced reflux-related residuals.^[9]^ Biochemically, a randomized stratified study by Forde et al reported significantly lower urinary allantoin (a marker of oxidative stress) in preterm infants receiving 1 hour of KMC compared with incubator care (*P* = .026), suggesting that reduced stress and inflammatory tone may also contribute to improved feeding tolerance over time.^[31]^ In parallel, oral-motor stimulation may promote maturation of suck–swallow–breathe coordination and cephalic-phase digestive responses, consistent with pooled evidence showing improvements in feeding efficiency and intake following oral-motor intervention.^[5]^

Clinically, these findings suggest a potentially actionable, workflow-compatible approach that may warrant consideration for supporting feeding trajectories in extremely preterm infants: consider integrating structured EOMI with KMC exposure delivered before the first oral-feeding training day, then use NOMAS severity at first training to help inform the intensity and monitoring of subsequent feeding advancement, while recognizing that prospective evaluation is needed to confirm effectiveness and guide implementation. A practical implementation pathway would include standardized nurse-delivered EOMI sessions during the tube-feeding phase, scheduled, documented KMC sessions with parents prior to the planned transition to oral-feeding training, and a bedside “feeding readiness and risk” huddle on the NOMAS assessment day to align the feeding plan with physiologic stability and predicted FI risk.^[1,2,32]^ Our risk-factor model suggests that infants with lower GA and birth weight, PDA, intrauterine infection, perinatal asphyxia, and especially those requiring invasive mechanical ventilation are at elevated FI risk^[33,34]^; these infants may benefit from anticipatory strategies such as more gradual feed advancement, tighter monitoring of intolerance signs, earlier lactation support to maximize human milk exposure, and deliberate coordination between respiratory-weaning plans and feeding transitions.^[10,35]^ The observed effect modification by GA group and mechanical ventilation implies that implementation should be stratified: more mature infants may realize larger marginal benefit from combined care, whereas ventilated infants may require adaptation (shorter, more frequent KMC sessions when stable, careful positioning to minimize abdominal distension, and explicit criteria for pausing feeds during respiratory decompensation).^[21,36]^ Importantly, the modest improvements seen in feeding efficiency and intake ratio suggest that incremental gains in oral transfer and daily intake may accumulate into earlier milestone achievement and fewer FI-related interruptions, which in turn may shorten parenteral nutrition exposure and may be associated with lower downstream complications in routine NICU pathways.^[37,38]^

This study has several strengths, including a relatively large extremely preterm sample, explicit temporal alignment of KMC exposure to occur on or before NOMAS assessment to support mediation ordering, prespecified model building with clinically interpretable covariates, and integrated reporting of both etiologic associations, mechanistic mediation (NOMAS pathway), and prognostic modeling for FI with discrimination and calibration. Nonetheless, important limitations remain. As a retrospective observational analysis, residual confounding and selection bias are unavoidable, particularly because KMC exposure likely reflects family availability, staff capacity, and infant stability.^[39,40]^ Some covariates (such as respiratory support, air pollution, and PDA) are illness markers that can vary over hospitalization, and time-varying confounding cannot be fully resolved without longitudinal causal methods.^[41]^ The time-to-milestone analyses were conducted among infants who achieved the milestones, which may introduce selection effects in interpretation.^[42,43]^ FI definitions vary across NICUs and reliance on charted residuals, vomiting, and abdominal girth can introduce measurement heterogeneity. Finally, NOMAS provides a pragmatic bedside assessment, but it does not capture all neurodevelopmental and sensorimotor dimensions relevant to feeding, which may explain why mediation was partial and why non-oral-motor pathways likely contributed to the remaining association.^[44,45]^

## 5. Conclusions

In this record-based retrospective observational study of extremely preterm infants, exposure to EOMI combined with KMC delivered on or before the first oral-feeding training day was associated with a consistent pattern of more favorable feeding outcomes, including lower odds of FI, a greater likelihood of reaching the prespecified 80% oral-intake milestone before discharge, shorter time to milestone attainment among achievers, and modestly higher feeding efficiency and intake ratio. Mediation analysis suggested partial mediation through early oral-motor function assessed by NOMAS severity, indicating that differences in NOMAS severity may account for a measurable portion of the observed association with FI, alongside additional pathways not captured by NOMAS. Independent risk factor modeling identified maturity and illness-severity markers as dominant correlates of FI and showed acceptable discrimination and calibration, supporting the potential utility of pragmatic risk stratification in routine care. Overall, these findings suggest that integrating structured early oral-motor stimulation with timely KMC may be a feasible approach to support feeding progression in extremely preterm infants, and they highlight the need for prospective studies with clearer temporal measurement and standardized feeding-intolerance phenotyping to strengthen causal inference and inform implementation.

## Acknowledgments

The authors would like to thank the clinical and nursing teams of Tangshan Maternal and Child Health Hospital for their support in patient care and data collection.

## Author contributions

**Conceptualization:** Shasha Cui.

**Data curation:** Kunyan Zhang.

**Formal analysis:** Kunyan Zhang.

**Funding acquisition:** Shasha Cui.

**Methodology:** Hui Yang.

**Writing – original draft:** Yue Chu, Tong Wang.

**Writing – review & editing:** Tong Wang.
